# A systematic review of the neuroprotective role and biomarker potential of GDF15 in neurodegeneration

**DOI:** 10.3389/fimmu.2024.1514518

**Published:** 2024-12-16

**Authors:** Finula I. Isik, Shannon Thomson, John F. Cueto, Jessica Spathos, Samuel N. Breit, Vicky W. W. Tsai, David A. Brown, Caitlin A. Finney

**Affiliations:** ^1^ Neuroinflammation Research Group, Centre for Immunology and Allergy Research, Westmead Institute for Medical Research, Sydney, NSW, Australia; ^2^ St. Vincent’s Centre for Applied Medical Research, St. Vincent’s Hospital and Faculty of Medicine, University of New South Wales, Sydney, NSW, Australia; ^3^ Westmead Clinical School, Faculty of Medicine and Health, University of Sydney, Sydney, NSW, Australia; ^4^ Western Sydney Local Health District, Institute for Clinical Pathology and Medical Research, NSW Health Pathology, Sydney, NSW, Australia; ^5^ School of Medical Sciences, Faculty of Medicine and Health, University of Sydney, Sydney, NSW, Australia

**Keywords:** GDF15, GFRAL, neurodegeneration, neurotrauma, inflammation, neurotrophic, biomarker

## Abstract

Neurodegeneration is characteristically multifaceted, with limited therapeutic options. One of the chief pathophysiological mechanisms driving these conditions is neuroinflammation, prompting increasing clinical interest in immunomodulatory agents. Growth differentiation factor 15 (GDF15; previously also called macrophage inhibitory cytokine-1 or MIC-1), an anti-inflammatory cytokine with established neurotrophic properties, has emerged as a promising therapeutic agent in recent decades. However, methodological challenges and the delayed identification of its specific receptor GFRAL have hindered research progress. This review systematically examines literature about GDF15 in neurodegenerative diseases and neurotrauma. The evidence collated in this review indicates that GDF15 expression is upregulated in response to neurodegenerative pathophysiology and increasing its levels in preclinical models typically improves outcomes. Key knowledge gaps are addressed for future investigations to foster a more comprehensive understanding of the neuroprotective effects elicited by GDF15.

## 1 Introduction

Neurodegenerative disorders, characterized by the gradual loss of neurons, have limited treatment options and present a significant diagnostic challenge in modern healthcare. These conditions result from a range of chronic diseases driven by complex neuropathogenic pathways or secondary to neurotrauma. Neuroinflammation is intimately associated with these events and can both mitigate and potentiate ongoing neuronal damage. On one hand, inflammatory mechanisms are integral in removing cellular debris and promoting repair and regeneration. On the other, chronic, inappropriately amplified, or suppressed responses, can exacerbate damage or propagate ongoing inflammatory cascades involved in generating neuropathology. Neuroinflammatory sequelae are now widely acknowledged to have an important role in responding to and/or promoting endogenous, neurodegenerative disease-specific protein aggregates including, but not limited to, α-synuclein, amyloid-β, hyperphosphorylated tau and more (reviewed in 1–3). Therefore, immunomodulation represents a promising avenue for the treatment of neurodegenerative conditions and are the basis of ongoing preclinical and clinical research. To date, however, most anti-inflammatory drugs fail to demonstrate significant, favorable therapeutic effects (4). These findings may be due to the suppression of key, beneficial inflammatory events, highlighting that ‘pro-inflammatory’ events at appropriate times may have a reparative rather than damaging immunomodulatory phenotype (4). Therefore, there is a pressing need to characterize the involvement of key inflammatory molecules in neurodegeneration and to identify novel therapeutic targets that can mitigate or prevent neurodegenerative processes in the central nervous system (CNS).

Growth differentiation factor 15 (GDF15; previously called macrophage inhibitory cytokine-1 MIC-1) is one such immunomodulatory factor that has been implicated in neurodegeneration. This GDNF family cytokine, within the transforming growth factor-β (TGF-β) superfamily, was first identified as a 25kDa disulfide-linked dimer (5). Like other TGF-β superfamily members, it is synthesized as a precursor protein that dimerizes and is processed by cleavage at the conserved RXXR sequence, resulting in the secretion of a mature, biologically active disulfide-linked dimer that rapidly diffuses into circulation. Unprocessed protein is sometimes secreted and binds to the extracellular matrix, possibly serving as a local reservoir (6). Centrally, GDF15 regulates non-homeostatic energy metabolism through its only known receptor, growth factor receptor α-like (GFRAL), expressed on neurons in the hindbrain area postrema (AP) and nucleus of the solitary tract (NTS). GDF15 and GFRAL form a complex with the REarranged during Transfection (RET) coreceptor to activate these hindbrain neurons (7–11). Until recently, GDF15 was incorrectly believed to act via classical TGF-β receptors I and II and SMAD pathways. Many *in vitro* studies demonstrating this effect used commercial recombinant GDF15 (rGDF15) stocks which are now known to be contaminated with TGF-β, rendering many conclusions related to the GDF15’s effects unreliable (12). Regardless, accumulating work utilizing transgenic mouse models indicate GDF15 can modulate peripheral immune cell infiltration and contribute to CNS/cardiac infarct or lesion healing (13–17). Moreover, this pleiotropic molecule is a well-established indicator of cellular stress. The GDF15 promotor has binding sites for many transcriptional regulators induced by cell stress, including p53 (18), EGR-1 (19), CHOP (20) and ATF4 (21). Elevated serum GDF15 is a feature of pregnancy and frequently observed in conditions like advanced cancers, chronic heart and renal failure, genetic mitochondrial diseases, obesity and type 2 diabetes, dementia and chronic inflammatory diseases (11), and is a reliable predictor of all-cause mortality (22, 23). Despite the first evidence of GDF15’s neuroprotective role reported over two decades ago (24), its role as a central or peripheral neurodegenerative biomarker and its potential neuroprotective effects remain poorly defined.

To address this gap in the literature and begin to elucidate the role of GDF15 in neurodegeneration, we performed a systematic review of studies examining this pleiotropic cytokine in neurodegenerative conditions. Key findings drawn from these studies were categorized into one of two main themes, which will be outlined in this present review: (1) investigating the neuroprotective role of GDF15 in animal and cell models of neurodegeneration, and (2), assessing GDF15 levels as a biomarker for neurodegenerative disease or injury. In doing so, we provide the first systematic examination of literature on GDF15 in relation to neurodegenerative physiopathology and highlight the potential for GDF15 as a viable therapeutic target across several neurodegenerative diseases.

## 2 Methods

### 2.1 Literature search

For the present systematic review, we employed a clear pipeline as previously detailed (25). In brief, we systematically searched three separate databases: PubMed, Scopus, and Web of Science. Our search terms included GDF15 and its synonyms (e.g. MIC-1) as well as terms related to the CNS (e.g. brain), as detailed in **Table 1**.

**Table 1:** Key terms for database search.

(“Growth differentiation factor 15” OR “GDF15” OR “macrophage inhibitory cytokine 1” OR “MIC-1” OR “prostate differentiation factor” OR “placental TGF-β” OR “PTGF β” OR non-steroidal anti-inflammatory drug activated gene-1” OR “NAG1” OR “placental bone morphogenic protein” OR “PLAB”) AND (“brain” OR “neuro*” OR “spinal” OR “nerv*”)

The search strategy incorporated multiple known names for GDF15 in combination with key nervous system terms.

*Truncation for term root to accommodate related terms.

The search terms were limited to title and abstract only and databases were searched on May 27, 2024. Following removal of duplicates, titles, abstracts, and full texts were reviewed and scored for inclusion by F.I. and S.T. based on eligibility criteria developed *a priori* (**Table 2**).

**Table 2:** Inclusion and exclusion selection criteria.

Inclusion criteria
**Subject** Male or female subjects Neuronal, glial, or mixed cell culture Brain, spinal cord, or nerve tissue Neurodegenerative injury or disease *in vitro* and/or *in vivo* **Treatment or variable** Administration of GDF15 cytokine Measurement of GDF15 levels Alteration of GDF15 levels *in vitro* and/or *in vivo* **Other** Study written in English Peer-reviewed, primary article
Exclusion criteria
Did not include the administration of GDF15 Did not measure levels of GDF15 Did not alter levels of GDF15 *in vitro* and/or *in vivo* Did not examine neurodegeneration or neurodegenerative outcomes Did not directly relate GDF15 to neurodegeneration or neurodegenerative outcome Not in English Not a primary study Could not locate full text Raised methodological concerns Not peer-reviewed

### 2.2 Eligibility criteria

Our goals in this systematic review were to assess GDF15’s involvement in neurodegenerative conditions to (1) elucidate the mechanisms that may underly its neuroprotective effects and (2) assess its suitability as a biomarker for neurodegenerative diseases. We therefore developed clear inclusion and exclusion criteria to address this (**Table 2**). Inclusion criteria included (1) a human, animal or cell model of neurodegenerative injury or insult, (2) confirmed neurodegenerative injury (*in vitro* or *in vivo*), (3) exogenous manipulation of GDF15 or direct measurement of GDF15, (4) study written in English and (5) a peer reviewed primary article. The studies were excluded if they did not fit these inclusion criteria, namely, they (1) did not include the measurement of, alteration of, or exogenous modulation of GDF15 levels *in vitro* and/or *in vivo*, (2) did not examine neurodegeneration or neurodegenerative outcomes, (3) did not directly relate GDF15 to neurodegeneration or neurodegenerative outcomes, (4) were not in English, (5) were not peer reviewed, (6) not a primary study, and/or (7) were limited by methodological concerns, including inadequate or inconsistent result reporting. We took a liberal approach to screening titles to prevent the inappropriate exclusion of full texts. Two assessors (F.I. and S.T.) were used in the screening of titles and abstracts, and wherever a discordance was identified, the citation defaulted to having its full text assessed. Although we aimed to include all relevant studies, some reporting biases may affect the overall findings and is a limitation of this review. This review was not registered.

## 3 Results

### 3.1 Literature search

**Figure 1:**
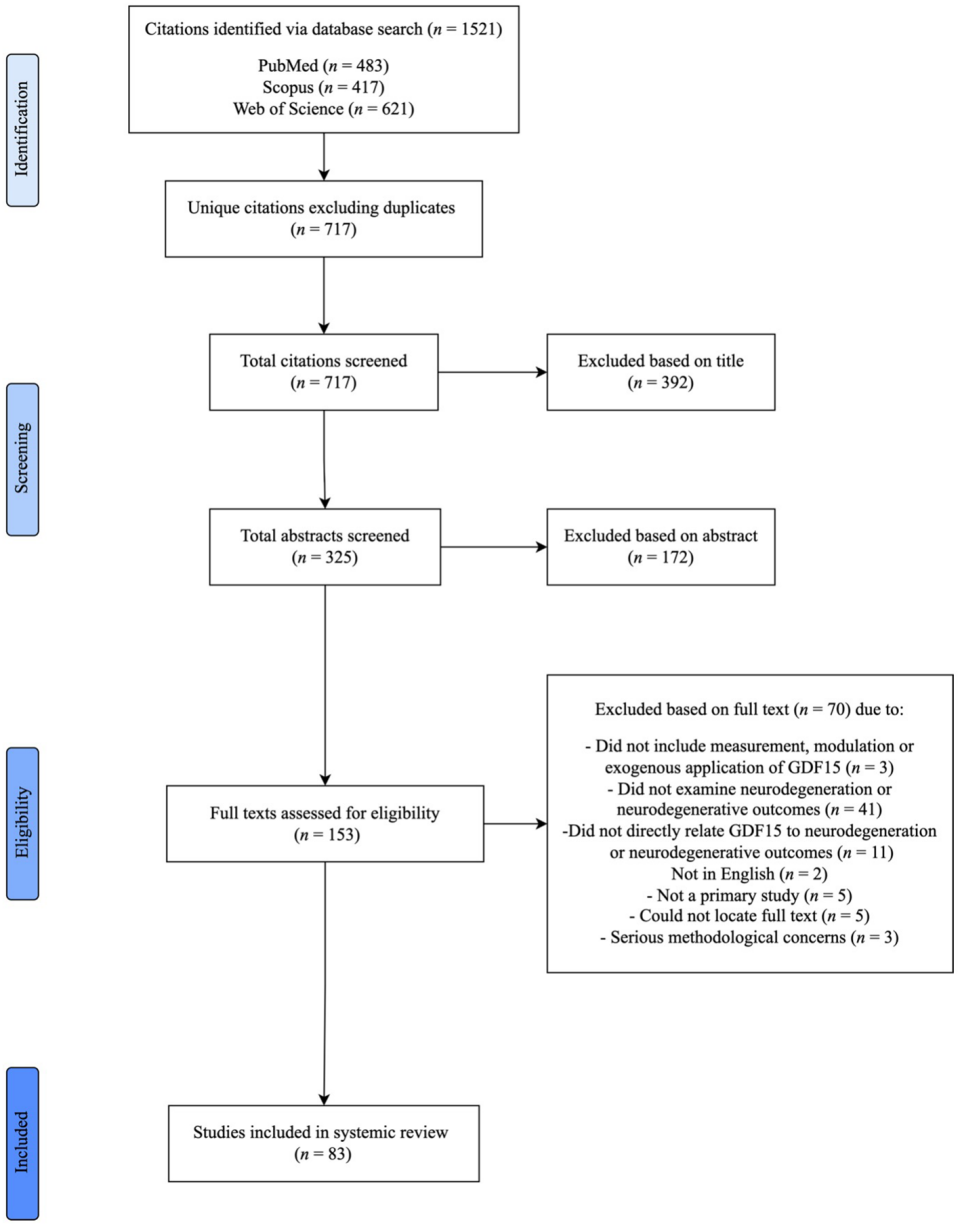
Systematic literature review process and result pipeline. PRISMA flow diagram highlighting the stages of literature identification, screening, exclusion, and inclusion to final full text.

The literature search conducted in PubMed, Scopus and Web of Science resulted in a total 1,521 citations. After removing duplicate citations, 717 remained. These citation titles and abstracts were then screened based on the eligibility criteria (**Table 2**). The interrater reliability indicated substantial agreement between both raters (91.77%, Cohen’s Kappa: 0.71). A total of 153 full texts were then reviewed for inclusion. Of these, 83 full texts met the eligibility criteria and were included in the review (**Figure 1**). The most common reason for exclusion was not examining neurodegeneration or neurodegenerative outcomes (n = 41). Publications were additionally excluded if they did not examine the relationship between neurodegenerative variables and GDF15 (n = 11). Other reasons for exclusion included studies that did not include the measurement, modulation, or exogenous application of GDF15 *in vivo* or *in vitro* (n = 3), were not in English (n = 2), were not a primary study (n = 5) and texts that could not be located despite our best efforts (n = 4). Three papers were excluded due to concerns related to inconsistencies in results reported in graphs, questioning the validity of the reported findings.

### 3.2 Characteristics of the included studies

The included studies examined GDF15 in humans (n = 52), animal models (n = 22), or cell culture models (n = 26). Several studies included a combination of subjects, including animal and cell (n = 11), human and cell (n = 3) and human and animal (n = 2), and all three subjects (n = 1). Extracted data was assessed qualitatively as the included studies were too heterogenous to group together or perform meta-analyses on.

In human studies (**Table 3**), GDF15 levels were measured in biological fluids (n = 50) and/or post-mortem CNS tissue (n = 3) of individuals affected by neurodegenerative disease or injury. This included mitochondrial disease (n = 14), Alzheimer’s disease (AD) or dementia (n = 13), synucleinopathy (n = 7), Multiple Sclerosis (MS) and/or Neuromyelitis Optica spectrum disorder (n = 7), glaucoma (n = 3), motor neuron diseases (MND) (n = 3), Charcot-Marie-Tooth disease (n = 2), Vanishing White Matter Disease (n = 1), stroke (n = 8) and subarachnoid haemorrhage (n = 1). Most studies (n = 33) involved cross-sectional analysis of GDF15 levels in blood, cerebrospinal fluid (CSF) or aqueous humor. The remaining studies (n = 18) were longitudinal. They either measured GDF15 levels across several timepoints, or related baseline GDF15 levels with follow-up neurological outcomes. One case study and one 2-sample Mendelian Randomization Study were also included. Full characteristics related to study design and participants are outlined below and in **Supplementary Tables 1**, **2**.

**Table 3:** Characteristics of human subjects.

Human Subject Characteristics	Number of studies featuring cohort
Disease	
Mitochondrial disease	14
Alzheimer’s disease (AD) and dementia	13
Synucleinopathy	7
Multiple Sclerosis (MS)/Neuromyelitis Optica Spectrum disorder (NMOSD)	7
Glaucoma	3
Motor neuron disease (MND)	3
Charcot-Marie-Tooth disease (CMT)	2
Vanishing White Matter disease (VWMD)	1
Stroke	8
Subarachnoid haemorrhage	1
GDF15 Measurement	
Serum	26
Plasma	18
Blood	2
CSF	3
Aqueous humour	3
Ex vivo tissue	3

For animal studies (**Table 4**), most used mice (n = 19), followed by rats (n = 5) and drosophila (n = 1). Several diseases were modelled, including AD (n = 3), Parkinson’s disease (PD) (n = 3), Charcot-Marie Tooth disease (n = 2), retinal degeneration and glaucoma (n = 3), Huntington’s disease, Spinal Muscular Atrophy, and Vanishing White Matter disease (n = 1, respectively). Studies also examined neurodegeneration secondary to induced CNS (n = 5) and peripheral NS (PNS) injury (n = 2), including spinal cord injury (SCI), cryolesion, excitotoxicity, stroke, and sciatic nerve crush. Cell culture studies (**Table 5**) used models derived from human (n = 16), mouse (n = 6), and rat (n = 10) origin. Most used neurons (n = 14), followed by astrocytes (n = 6), mixed midbrain cultures (n = 4), microglia (n = 3), brain-derived endothelia (n =1), retinal ganglion cells (n = 1) and Schwann cells (n =1). Patient derived fibroblasts (n = 2), mesenchymal stem cells (n = 2), neural stem cells (n = 1) and patient derived mesenchymal stromal cells (n = 1) were also used. Like animal studies, neurodegenerative disease was induced in these cells to model AD (n = 4), PD (n = 5), Amyotrophic Lateral Sclerosis (ALS), Charcot-Marie Tooth disease, Huntington’s disease, MS, retinal degeneration, Spinal Muscular Atrophy and Vanishing White Matter disease (n = 1, respectively). The remaining studies induced cellular injury (n = 9), via neuroinflammation, low potassium, ferroptosis, excitotoxicity, mitochondrial dysfunction, or DNA damage. GDF15 secretion and expression was measured in cell and animal models of neurodegeneration (n = 26). Full characteristics of *in vitro* and *in vivo* studies are outlined in **Supplementary Table 3**.

**Table 4:** Characteristics of animal subjects.

Animal Characteristics	Number of studies featuring animal
Species, strain	
Mouse, all	19
Rat, all	5
Drosophila, HTT93Q exon1	1
Neurodegenerative Disease/Condition Model	
AD	3
Parkinson’s disease (PD)	3
Retinal degeneration (RD)/Glaucoma	4
Huntington’s disease (HD)	1
Spinal Muscular Atrophy Jokela (SMAj)	1
Vanishing White Matter disease (VWMD)	1
CNS Injury	5
PNS Injury	2

**Table 5:** Characteristics of Cell Culture subjects.

Cell Culture Characteristics	Number of studies featuring cell
Cell origin	
Human	16
Mouse	6
Rat	10
Neurodegenerative Disease/Condition Model	
Alzheimer’s disease (AD)	5
Parkinson’s disease (PD)	5
Amyotrophic Lateral Sclerosis (ALS)	2
Charcot-Marie-Tooth disease (CMT)	1
Huntington’s disease (HD)	1
Multiple Sclerosis (MS)	1
Retinal degeneration (RD)/Glaucoma	1
Spinal Muscular Atrophy Jokela (SMAj)	1
Vanishing White Matter disease (VWMD)	1
CNS injury (all)	9

### 3.3 GDF15 is involved in neuroprotective molecular mechanisms in neurodegeneration

The animal and cell culture studies included in this systematic review highlight GDF15’s neuroprotective role across a wide range of pathophysiological mechanisms. In general, they showed that increasing GDF15 was beneficial, whilst decreasing its levels was detrimental. It should be noted that many of these studies used commercial rGDF15 protein, which is known to be a source of TGF-β contamination (12). This makes it difficult to solely attribute these protective effects to our cytokine of interest. Although we have included details of these flagged studies (see **Tables 6**–**8**), we only discuss the results of studies that are not affected by this issue.

**Table 6:** Modulating GDF15 in ND models is neuroprotective by promoting cell survival.

How GDF15 relates to pathophysiology	Neurodegenerative Condition: Model	GDF15 Modulation (methodology)	Effect of GDF15 Modulation	Experimental support (Yes/No/-)	Ref
**Prevents apoptosis**	RD: Mouse, optic nerve crush	Decrease (GDF15 KO vs Wt)	No change in Atf, Bad, Bcl-2	-	(26)
	RD: Mouse, optic nerve crush	Decrease (GDF15 KO vs Wt)	Increased galanin (3 dpi), caspase-8 (7 dpi)	Yes	
	CMT: RT4+WJ-MSC coculture, PMP22 overexpression	Increase (no methodology)	Decreased PI cells %	Yes	(27)
	PD: SH-SY5Y, rotenone	Increase (GDF15 plasmid)	Increased Bcl-2/BAX, PGC1-a, TH	Yes	(28)
	PD: SH-SY5Y, rotenone	Increase (GDF15 plasmid)	Decreased P53	Yes	
	PD: SH-SY5Y, rotenone	Increase (GDF15 plasmid)	Reduced TUNEL+ cells	Yes	
	CNS Injury: Primary cerebellar granule cells, low K+	Increase (0.1-100 ng/mL rGDF15 application, purified in-house stock)	Decreased release of lactate dehydrogenase, PI cells % and TUNEL+ cells	Yes	(24)
	CNS Injury: Primary cerebellar granule cells, low K+	Increase (0.1-100 ng/mL rGDF15 application, purified in-house stock)	Decreased active ERK, decreased P-c-Jun	Yes	
	CNS Injury: Primary cerebellar granule cells, low K+	Increase (0.1-100 ng/mL rGDF15 application, purified in-house stock)	Increased active P-Akt/GSK-3	Yes	
	AD: SH-SY5Y+siBMC-derived exosome, Aβ42	Decrease (siGDF15 in BMSCs)	Increased PI cell %	Yes	(29)
	AD: SH-SY5Y+siBMC-derived exosome, Aβ42	Decrease (siGDF15 in BMSCs)	Increased Bad, Bax, caspase-3, decreased Bcl-2	Yes	
	AD: SH-SY5Y+siBMC-derived exosome, Aβ42	Increase (rGDF15 application, no methodology)	Decreased Bad, Bax, caspase-3, increased Bcl-2	Yes	
	AD: SH-SY5Y, Aβ44	Increase (GDF15 plasmid)	Decreased PI cells %	Yes	(30)
	AD: SH-SY5Y, Aβ45	Increase (GDF15 plasmid)	Decreased Bax, Bad, caspase-3, increased Bcl-2	Yes	
**Promotes cell viability**	PD: SH-SY5Y, rotenone	Increase (0-100ng/mL rGDF15 application, R&D Systems)	Increased cell viability %	Yes	(28)
	PD: HT22, oligomycin	Increase (GDF15 plasmid)	Increased cell viability %	Yes	(31)
	AD: SH-SY5Y+siBMC-derived exosome, Aβ42	Decrease (siGDF15 in BMSCs)	Decreased cell viability %	Yes	(29)
**Promotes cell proliferation**	AD: NSC+hUCB-MSC coculture	Decrease (GDF15 siRNA in hUCB-MSCs)	Decreased Sox2, nestin	Yes	(32)
	AD: NSC+hUCB-MSC coculture	Increase (20 ng/mL rGDF15 application, no source)	Increased Sox2, nestin, PCNA	Yes	
	AD: NSC+hUCB-MSC coculture	Increase (20 ng/mL rGDF15 application, no source)	Increased BrdU+, NeuN+	Yes	
	AD: Mouse, APP/PS1	Increase (0.5 µg/kg rGDF15 bilateral dentate gyri injection, no source)	Increased #hippocampal NSCs	Yes	
	AD: Mouse, APP/PS1	Increase (1 µg/kg rGDF15 bilateral dentate gyri injection, no source)	Decrease #hippocampal NSCs	No	
	PD: Primary midbrain culture, iron	Increase (0.01-10ng/mL rGDF15 application, purified in-house stock)	No change to BrdU+	-	(33)
**Promotes pathogenic protein clearance**	AD: BV2, Aβ42	Increase (100ng/mL rGDF15 application, R&D Systems)	Decreased Aβ42 secretion	Yes	(34)
	AD: Aβ42 treated BV2+hUCB-MSC coculture	Decrease (GDF15 siRNA in hUCB-MSCs)	Increased Aβ42 secretion	Yes	
	AD: Mouse, 5xFAD	Increase (1 µg/kg rGDF15 bilateral dentate hippcampi, R&D Systems)	Decreased Aβ42 secretion	Yes	
	AD: Mouse, 5xFAD	Increase (1 µg/kg rGDF15 bilateral dentate hippocampi, R&D Systems)	Decreased Aβ42 plaque area	Yes	
	PD: SH-SY5Y, rotenone	Increase (GDF15 plasmid)	Decreased α-syn mRNA	Yes	(28)
**Induces degrading enzymes**	AD: BV2, Aβ42	Increase (100 ng/mL rGDF15 application, R&D Systems)	Increased IDE	Yes	(34)
	AD: BV2, Aβ42	Increase (100 ng/mL rGDF15 application, R&D Systems)	No change NEP	-	
	AD: Mouse, 5xFAD	Increase (1 µg/kg rGDF15 bilateral dentate hippocampi, R&D Systems)	Increased IDE	Yes	
**Promotes synaptic activity**	AD: Primary hippocampal neurons	Increase (20 ng/mL rGDF15 application, no source)	Increased action potential stimulation	Yes	(32)
**Recovers mitochondrial dysfunction**	PD: HT22, oligomycin	Increase (GDF15 plasmid)	Increased mitochondrial membrane potential	Yes	(31)
	AD: patient fibroblasts	Decrease (GDF15 siRNA)	Decreased SDHA, UQCRC2 and ATP5PD mitochondrial complex subunits	Yes	(35)
**Inhibits nitric oxide**	PD: Primary neuron-glia and neuron enriched midbrain culture from GDF15 KO and Wt mice, 6-OHDA	Decrease (GDF15 KO vs Wt mice)	No change NO levels	-	(36)
	PD: Primary microglia, IFN-γ	Increase (100ng/mL rGDF15 application, no source)	No change NO levels	-	
**Prevents oxidative stress**	AD: SH-SY5Y, Aβ42	Increase (GDF15 plasmid)	Increased SIRT1, Nrf-2, HO-1	Yes	(30)
	AD: SH-SY5Y, Aβ42	Increase (GDF15 plasmid)	Increased SOD and GSH	Yes	
**Promotes oxygen consumption**	PD: HT22, oligomycin	Increase (GDF15 plasmid)	Increased oxygen consumption rate	Yes	(31)
**Inhibits ROS levels and activity**	PD: SH-SY5Y, rotenone	Increase (GDF15 plasmid)	Reduced intracellular and mitochondrial ROS	Yes	(28)
	CNS Injury: Primary cerebellar granule cells, low K+	Increase (0.1-100 ng/mL rGDF15 application, purified in-house stock)	Decreased ROS	Yes	(24)
	AD: SH-SY5Y, Aβ42	Increase (GDF15 plasmid)	Decreased ROS and MDA	Yes	(30)

Green indicates beneficial effect of increased GDF15, red indicates detrimental effect of increased GDF15, and grey indicates no effect of increased GDF15. Yellow indicates where a study used rGDF15 with potential unknown contaminants.

**Table 7:** Modulating GDF15 in ND models is neuroprotective by promoting cellular regeneration and functional recovery.

How GDF15 relates to pathophysiology	Neurodegenerative Condition: Model	GDF15 Modulation (methodology)	Effect of GDF15 Modulation	Experimental support (Yes/No/-)	Ref
**Prevents neuron loss**	RD: Mouse, optic nerve crush	Decrease (GDF15 KO vs Wt)	No change in #RGC, DAergic or ChAT neurons	-	(26)
	RD: Primary retinal ganglion cell	Increase (0.1, 1 µg/mL rGDF15 application, Aviscera Bioscience)	No change increase #RGC	-	(37)
	RD: Mouse, optic nerve crush	Increase (100 µg/mL rGDF15 intravitreal injection, Aviscera Bioscience)	Increased #RGCs (10 dpi)	Yes	
	AD: Mouse, APP/PS1	Increase (0.5 µg/kg rGDF15 bilateral dentate gyri injection, no source)	Increased #mature neurons	Yes	(32)
	AD: Mouse, APP/PS1	Increase (1 µg/kg rGDF15 bilateral dentate gyri injection, no source)	Increased #mature neurons	Yes	
	PD: Primary VTA/SN mixed culture, MPP+	Increase (50-200 ng/mL rGDF15 application, no source)	Increased surviving #DAergic neurons	Yes	(38)
	PD: Primary VTA/SN mixed culture, MPP+	Decrease (GDF15 siRNA in VTA astrocytes)	Decreased surviving #DAergic neurons	Yes	
	PD: iPSC-derived DAergic neurons, MPP+	Increase (50-400 ng/mL rGDF15 application, no source)	Increased surviving #DAergic neurons	Yes	
	PD: Mouse, MPTP	Decrease (GDF15 KO vs Wt)	No change to #DAergic neurons	-	(39)
	PD: Primary neuron-glia, and neuron enriched midbrain culture from GDF15 KO and Wt mice, 6-OHDA	Decrease (GDF15 KO vs Wt mice)	Decreased #DAergic neurons	Yes	(36)
	PD: Primary neuron-glia midbrain culture from GDF15 KO and Wt mice, 6-OHDA	Increase (100ng/mL rGDF15 application, no source)	No change to #DAergic neurons	-	
	PD: Primary neuron enriched midbrain culture from GDF15 KO and Wt mice, 6-OHDA	Increase (100ng/mL rGDF15 application, no source)	Increased #DAergic neurons	Yes	
	PD: Mouse, 6-OHDA	Decrease (GDF15 KO vs Wt)	Decreased #DAergic neurons	Yes	
	SMA: iPSC-derived motoneurons, growth-factor deprivation	Increase (10-200 ng/mL rGDF15 application, Peprotech)	Increased #motoneurons	Yes	(40)
	PD: Primary midbrain culture, iron	Increase (0.01-10ng/mL rGDF15 application, purified in-house stock)	Increased #DAergic neurons	Yes	(33)
	PD: Rat, 6-OHDA	Increase (10, 40 µg rGDF15 unilateral SN/LV injection, purified in-house stock)	Increased #DAergic neurons	Yes	
	CNS Injury: Mouse, aged	Decrease (GDF15 KO vs Wt)	Decreased #motoneruons and DRG neurons	Yes	(41)
	CNS Injury: Mouse, facial nerve transection	Increase (5 µg, rGDF15 via gel foam, R&D Systems and Biopharm)	Increased #motoneurons	Yes	
	HD: Drosophila HTT93Q exon1 mut	Decrease (GDF15/maverick RNAi)	Increased #photoreceptors	No	(42)
	PNS Injury: Mouse, sciatic nerve lesion	Decrease (GDF15 KO vs Wt)	No change in #DRG neurons	-	(43)
	ALS: spinal motoneurons from SOD1^G93A^ and Wt mice, neurotrophic factor deprivation	Increase (10-100 ng/mL rGDF15 application, R&D Systems)	No change in % surviving motoneurons	-	(44)
	ALS: spinal motoneurons from SOD1^G93A^ and Wt mice, NO donor DETANONOate	Increase (10 ng/mL rGDF15 application, R&D Systems)	Increased % surviving motoneurons	Yes	
**Promotes axon regeneration**	PNS Injury: Mouse, sciatic nerve lesion	Decrease (GDF15 KO vs Wt)	No change #axons	-	(43)
**Promotes myelination**	CMT: RT4+WJ-MSC coculture, PMP22 overexpression	Increase (no methodology)	Increased Oct6, MPZ	Yes	(27)
	CMT: Mouse, Pmp22Tr-J/J	Increase (1 µg/kg intramuscular injection rGDF15, R&D Systems)	Decreased #demyelinating/dysmyelinating sciatic nerves	Yes	
	PNS Injury: Mouse, sciatic nerve lesion	Decrease (GDF15 KO vs Wt)	No change in g-ratio in lesioned side, decrease in non-lesioned side	-	(43)
**Promotes neurite outgrowth and/or axon lengthening**	RD: Primary retinal ganglion cell	Increase (0.1, 1 µg/mL rGDF15 application, Aviscera Bioscience)	Increased neurite and axon length	Yes	(37)
	PD: Primary neuron-glia and neuron enriched midbrain culture from GDF15 KO and Wt mice, 6-OHDA	Decrease (GDF15 KO vs Wt mice)	Decreased neurite length	Yes	(36)
	PD: Primary neuron-glia and neuron enriched midbrain culture from GDF15 KO and Wt mice, 6-OHDA	Increase (100ng/mL rGDF15 application, no source)	No change to neurite length	-	
	CNS Injury: Mouse, SCI	Increase (GDF15 Tg vs Wt, KO)	Increased spared tissue (28 dpi)	Yes	(15)
	CNS Injury: Mouse, SCI	Decrease (GDF15 KO vs Wt, Tg)	No change to spared tissue	-	
**Promotes functional recovery (motor/sensory)**	CNS Injury: Mouse, SCI	Increase (GDF15 Tg vs Wt, KO)	Increased BMS (7dpi onwards)	Yes	(15)
	CNS Injury: Mouse, SCI	Decrease (GDF15 KO vs Wt, Tg)	No change in BMS	-	
	CNS Injury: Mouse, aged	Decrease (GDF15 KO vs Wt)	Decreased rotarod performance	Yes	(41)
	PD: Rat, 6-OHDA	Increase (40 µg rGDF15 unilateral SN/LV injection, purified in-house stock)	Decreased rotational asymmetry	Yes	(33)
	PD: Rat, 6-OHDA	Increase (10 µg rGDF15 unilateral SN/LV injection, purified in-house stock)	Decreased amphetamine-induced rotations	Yes	
	PNS Injury: Mouse, sciatic nerve lesion	Decrease (GDF15 KO vs Wt)	No change in conduction velocity or amplitude (5 and 9 weeks post injury)	-	(43)
	PNS Injury: Rat, sciatic nerve injury	Increase (10ng/mL rGDF15 via hydrogel, R&D Systems)	Partial increase in SSI, CMAP and decreased axon loss % (11 wks post injury)	Yes	(45)
	PNS Injury: Rat, sciatic nerve injury	Increase (10ng/mL rGDF15 via hydrogel, R&D Systems)	Increased pinch reflex (21 dpi)	Yes	

Green indicates beneficial effect of increased GDF15, red indicates detrimental effect of increased GDF15, and grey indicates no effect of increased GDF15. Yellow indicates where a study used rGDF15 with potential unknown contaminants.

**Table 8:** Modulating GDF15 in ND models is neuroprotective by mediating inflammation.

How GDF15 relates to pathophysiology	Neurodegenerative Condition: Model	GDF15 Modulation (methodology/origin)	Effect of GDF15 Modulation	Experimental support (Yes/No/-)	Ref
**Promotes astrogliosis**	PD: Mouse, MPTP	Decrease (GDF15 KO vs Wt)	No change in astrocytic reaction	-	(39)
	PD: Primary neuron-glia and neuron enriched midbrain culture from GDF15 KO and Wt mice, 6-OHDA	Decrease (GDF15 KO vs Wt mice)	Decreased #astrocytes	Yes	(36)
	PD: Primary midbrain culture, iron	Increase (0.01-10 ng/mL rGDF15 application, purified in-house stock)	No change to #astrocytes	-	(33)
**Promotes microglia proliferation/activation**	CNS Injury: Mouse, SCI	Increase (GDF15 Tg vs Wt, KO)	No change in microglia %	-	(15)
	CNS Injury: Mouse, SCI	Decrease (GDF15 KO vs Wt, Tg)	No change in microglia %	-	
	CNS Injury: Mouse, aged	Decrease (GDF15 KO vs Wt)	No change in microglial activation	-	(41)
	PD: Mouse, MPTP	Decrease (GDF15 KO vs Wt)	No change in microglia activation	-	(39)
	PD: Primary neuron-glia and neuron enriched midbrain culture from GDF15 KO and Wt mice, 6-OHDA	Decrease (GDF15 KO vs Wt mice)	No change in microglial activation	-	(36)
	PD: Primary microglia, IFN-γ	Increase (100 ng/mL rGDF15 application, no source)	No change in microglial activation	-	
	PD: Mouse, 6-OHDA	Decrease (GDF15 KO vs Wt)	Decreased #microglia	Yes	
**Promotes immune cell infiltration**	CNS Injury Mouse, SCI	Increase (GDF15 Tg vs Wt, KO)	Increased leukocytes (28 dpi)	Yes	(15)
	CNS Injury Mouse, SCI	Increase (GDF15 Tg vs Wt, KO)	Increased macrophages, DCs, T-cells (7 dpi onwards)	Yes	
	CNS Injury Mouse, SCI	Decrease (GDF15 KO vs Wt, Tg)	No change in leukocytes and macrophages	-	
	CNS Injury Mouse, SCI	Decrease (GDF15 KO vs Wt, Tg)	Decreased DCs, T-cells (28 dpi)	Yes	
	PNS Injury: Mouse, sciatic nerve lesion	Decrease (GDF15 KO vs Wt)	Increased #macrophages (7, 14 dpi)	Yes	(43)
**Alters cytokine expression profile**	PNS Injury: Mouse, sciatic nerve lesion	Decrease (GDF15 KO vs Wt)	No change CCL2 (0 – 14 dpi; nerve tissue distal to crush)	-	(43)
	PNS Injury: Mouse, sciatic nerve lesion	Decrease (GDF15 KO vs Wt)	Increased IL-1β (0.5 - 7 dpi), IL-6 (3, 7 dpi), MAC-2 (0, 1, 7 dpi), Arg-1 and Ym-1 (1, 3 dpi; nerve tissue distal to crush)	Yes	
	CNS Injury (SCI)	Increase (GDF15 Tg vs Wt, KO)	Increased CCL2 (28 dpi; injured SC)	Yes	(15)
	CNS Injury (SCI)	Increase (GDF15 Tg vs Wt, KO)	No change in IL-6, IL-12p70, TNFa, IFNy, IL10 (28 dpi; injured SC)	-	
	PD: Mouse, MPTP	Decrease (GDF15 KO vs Wt)	Decreased iNos, TNFα, IL-6, Arg1, Fizz-1, Ym1 (90 dpi; CPu and SN)	Yes	(39)
	PD: Mouse, MPTP	Decrease (GDF15 KO vs Wt)	Increased TGFB-1 (4 dpi; CPu), decreased TGFB-1 (90 dpi; CPu and SN)	Yes	
	PD: Mouse, 6-OHDA	Decrease (GDF15 KO vs Wt)	No change in TGFβ-1, TNF-α	-	(36)
	PD: Mouse, 6-OHDA	Decrease (GDF15 KO vs Wt)	Increased IL-6 (6.5 dpi; CPu) and iNOS (14 dpi; SN)	Yes	
	PD: Primary neuron enriched midbrain culture from GDF15 KO and Wt mice, 6-OHDA	Decrease (GDF15 KO vs Wt mice)	No change in TNF-α, IL-6	-	
	PD: Primary microglia, IFN-γ	Increase (100ng/mL rGDF15 application, no source)	No change in TNF-α, IL-6	-	
	PD: Primary neuron-glia, midbrain culture from GDF15 KO and Wt mice, 6-OHDA	Decrease (GDF15 KO vs Wt mice)	No change in TNF-α	-	
	PD: Primary neuron-glia, midbrain culture from GDF15 KO and Wt mice, 6-OHDA	Decrease (GDF15 KO vs Wt mice)	Increased expression IL-6	Yes	
	AD: SH-SY5Y+siBMC-derived exosome, Aβ42	Decrease (siGDF15 in BMSCs)	Increased TNFα, IL-6, IL-1B, IL-8	Yes	(29)
	AD: SH-SY5Y+siBMC-derived exosome, Aβ42	Increase (rGDF15 application, no methodology)	Decreased TNFα, IL-6, IL-1B, IL-8	Yes	
	AD: SH-SY5Y, Aβ42	Increase (GDF15 plasmid)	Decrease TNFα, IL-6, IL-1B, IL-8	Yes	(30)

Green indicates beneficial effect of increased GDF15, red indicates detrimental effect of increased GDF15, and grey indicates no effect of increased GDF15. Yellow indicates where a study used rGDF15 with potential unknown contaminants.

Overall, the studies examining GDF15 in neurodegenerative models highlight its role in minimizing induced cell death (**Table 6**). Specifically, apoptosis was repeatedly demonstrated to be mitigated by GDF15 modulation. Increasing GDF15 levels *in vitro* decreased the proportion of PI positive (24, 30) and TUNEL positive cells (24, 28), as well as reduced DNA fragmentation caused by apoptotic damage. Further, anti-apoptotic genes Bcl-2, PGCI-α and TH were increased whilst pro-apoptotic Bad, Bax, caspase-3 and P53 were decreased (28, 30). Reciprocal findings were demonstrated with knockdown of GDF15 in culture (29) and GDF15 knockout mice (26). The anti-apoptotic effects of GDF15 were mediated by activating the PI3K/Akt pathway and blocking ERK activation. The protective effect of GDF15 on cerebellar granule cells exposed to low potassium was ablated by selective PI3K inhibitors LY294002 and wortmannin (24). Additionally, low potassium induced ERK and in turn c-Jun activation but was prevented by GDF15 (24). A more recent publication supported this by highlighting that the PI3K/Akt signalling pathway was significantly enriched in oligomycin treated neurons that overexpressed GDF15 (31). As GDF15 is known to phosphorylate Erk1/2 *in vivo* (46), it is therefore likely that a neuroprotective effect of GDF15 occurs via this pathway.

Previous reports highlight GDF15-GFRAL binding initiates RET signalling cascades including activation of Akt, Fos, Erk1/2 and phospholipase C (11), however, only three studies in this present review directly examined this receptor and its signalling cascade. GFRAL expression was identified in dopaminergic neurons (38), primary retinal ganglion cells (37), and in mouse retina and optic nerve (37), although, expression in these regions has not been identified in other publications. Transcriptomic analysis of wildtype and ALS SOD1^G93A^ mice failed to show GFRAL expression in spinal cord tissue (44). The neurotrophic effect of the GDF15-GFRAL-RET complex was demonstrated in retinal ganglion cells using RET inhibition. Specifically, rGDF15 activated Akt and promoted neurite and axon length in these cells. This effect was suppressed to the level of vehicle-control when co-treated with selective RET inhibitor GSK317910, which was also shown to suppress Akt phosphorylation (37). Though this study used rGDF15 derived from *E. coli*, which may have residual endotoxin contamination or may have been misfolded, it provides intriguing support for further investigation. Further work utilizing purified rGDF15 should be undertaken to confirm the mechanism of GDF15-GFRAL RET receptor-ligand interactions in Akt-mediated cell survival responses.

In addition to anti-apoptotic activity, GDF15 further promotes neuronal survival by increasing cell viability (**Table 6**). One study highlighted that genetically increasing GDF15 improved viability of HT22 cells in response to mitochondrial toxin oligomycin (31). Another study reinforced this by examining the effect of incubating cells with exosomes extracted from siGDF15 transfected mesenchymal stem cells (MSCs). When co-cultured with SH-SY5Y cells, these exosomes potentiated Aβ-induced loss of cell viability (29). In addition, GDF15 was shown to regulate adult neural stem cell (NSC) proliferation. Normally, MSCs boost NSC proliferation through secretion of transcription factors Sox2 and nestin, an effect that is inhibited in siGDF15 MSC transfected co-cultures (32). However, a different study showed the application of rGDF15 failed to increase BrdU+ staining in iron-treated midbrain cultures (33). Therefore, while GDF15 may be neurotrophic, there is insufficient evidence that supplementing exogenously can increase neuronal proliferation following injury.

GDF15 also has a positive impact on bioenergetic stress following injury and insult (**Table 6**). One study showed that reducing GDF15 in AD patient-derived fibroblasts led to a decrease in expression of mitochondrial complex subunits SDHA, UQCRC2 and ATP5PD (35). Other publications highlighted that increasing GDF15 levels *in vitro* helped restore mitochondrial function and reduced oxidative stress. Specifically, GDF15 plasmid transfection of neurons attenuated mitochondrial injury by restoring mitochondrial membrane potential and oxygen consumption (31), reducing ROS levels (24, 28, 30), and increasing expression of antioxidants SOD and GSH in response to Aβ42 (30). This in turn promotes cell survival by inhibiting ROS-induced activation of pro-apoptotic ERK pathways (24).

### 3.4 GDF15 may promote functional recovery by attenuating neuron loss and damage

Modulation of GDF15 in the included studies was partially associated with improved functional outcomes in neurodegenerative models (**Table 7**). An early study by Strelau and colleagues demonstrated functional motor recovery could be promoted in 6-OHDA treated rats via unilateral injection of rGDF15 into substantia nigra/lateral ventricle. Higher doses decreased rotational asymmetry, and low doses decreased amphetamine-induced rotations (33). Complementing this, GDF15 knockout mice were shown to have poorer rotarod performance (41). GDF15 overexpressing mice with SCI have improved motor scores from 7 days post injury (dpi) onwards, although the GDF15 null mice did not differ from the wildtype (15). Similarly, GDF15 knockout genotype did not influence electromyography measures of nerve conductivity after sciatic nerve lesion (43), highlighting that while increased GDF15 improved motor performance following injury, a lack of GDF15 did not necessarily impede recovery.

The above studies attributed changes in functional outcomes following induced injury/insult to the extent of neuron loss, however, the evidence linking this to GDF15 is mixed (**Table 7**). In support of GDF15’s involvement, aged GDF15 null mice have fewer motoneurons and dorsal root ganglia neurons than their wildtype counterparts (41). Another study showed loss of dopaminergic neurons and decreased neurite length following 6-OHDA treatment in GDF15 knockout mice and their midbrain neuron-glia cultures relative to wildtype (36). rGDF15, however, rescued dopaminergic neurons in 6-OHDA treated rats and iron-treated midbrain cultures (33). Furthermore, GDF15 overexpression in SCI mice decreased the area of injured spinal cord tissue at 28 dpi (15). However, the remaining studies knocking down or silencing GDF15 do not consistently replicate these effects. In fact, one study showed silencing GDF15 was protective in a drosophila model of Huntington’s disease, by ameliorating endoplasmic reticulum-stress induced apoptosis in photoreceptors (42). In mice, neuron loss did not vary between knockout and wildtype genotypes following sciatic nerve lesion (43), optic nerve crush (26), or MPTP treatment (39). Further, GDF15 knockout had no effects on injury size (15), axon number, or g-ratio as a measure of demyelination (43). Interestingly, when grown in a co-culture, GDF15 silencing of VTA astrocytes potentiated dopaminergic neuron loss after MPP+ treatment (38). This suggests that although astrocytes may contribute to GDF15-mediated neuron preservation in mixed neuron-glia culture, compensatory mechanisms *in vivo* may offset the effects of GDF15 silencing, explaining the lack of exacerbated neuron loss. Further research would benefit from replicating these models using transgenic or pharmacological GDF15 expression to fully uncover its protective effect.

### 3.5 GDF15 mediates peripheral rather than local neuroinflammatory responses

Multiple studies included in this review examined the effect of GDF15 on inflammation and immune responses in neurodegenerative models (**Table 8**). First and foremost, several studies indicated changes in cytokine expression in response to GDF15 modulation. GDF15 overexpressing mice with SCI showed significantly increased CCL2 expression, but not IL-6 at 28 dpi (15). Contrastingly, while GDF15 knockout showed no change in CCL2, IL-6 and other signature molecules IL-1β, MAC-2, Arg-1 and Ym1 were elevated in the first 0 – 7 days in nerve tissue distal to crush injury (43). Another study found 6-OHDA-treated GDF15 knockout mice had increased IL-6 and iNos at 6 and 14 dpi in the caudate putamen and substantia nigra respectively, with no change in TGF-β1 or TNF-α (36). However, limited changes were identified following MPTP injection. Cytokines iNos, TNFα, IL-6, Arg1, Fizz-1, Ym-1 and TGFβ-1 were unchanged between GDF15 null and wildtype mice until 90 dpi, when they were downregulated (39). This milder cytokine response could be linked to the relatively transient nature of MPTP neurotoxicity (39), however, without consistent timepoints between different models, this is speculative. Nevertheless, GDF15’s role in altering cytokine expression following neurodegenerative injury or insult was further indicated by *in vitro* studies. Following 6-OHDA treatment, IL-6 was more elevated in primary mixed cultures derived from GDF15 knockout mice (36). SH-SY5Y neurons treated with Aβ42 expressed less TNFα, IL-6, IL-1B and IL-8 when GDF15 was overexpressed (30). When exposed to siGDF15 treated MSC-derived exosomes, the opposite effect was observed in these neurons (29). Overall, although modulation of GDF15 alters cytokine expression profiles in response to induced neurodegeneration, lack of replicability in the types of cytokines and the timepoints measured between these studies makes an overall pattern difficult to ascertain.

Secondary to cytokine dysregulation, our review identified two *in vivo* studies that examined how GDF15 may influence peripheral immune infiltration in response to induced injury mechanisms (**Table 8**). One study identified an altered immune cell response in GDF15 transgenic and knockout mice following SCI. Macrophages, dendritic cells, and T-cells were elevated from 7 dpi, and leukocytes from day 28 in the injured spinal cord of GDF15 overexpressing mice. GDF15 null mice however, had reduced dendritic and T-cell infiltration, and unchanged macrophage and leukocyte levels (15). The other study examined responses in the PNS, where GDF15 knockout increased macrophages distal to the injury in the 1 – 2 weeks following sciatic nerve lesion (43). As macrophage recruitment was increased in one GDF15 null injury model and not the other, these differences may relate to inherent distinctions between the peripheral and central nervous system, though further experimental work is required to support this assertion.

Neuroinflammation underlying neurodegeneration is associated with glial activity and proliferation. In fact, two included studies showed that astrocytes express and secrete GDF15 in response to induced neuroinflammation (47, 48). However, only one of our included studies demonstrated an effect of modulating GDF15 on these responses (**Table 8**). 6-OHDA treated-GDF15 knockout mice had reduced numbers of microglia (36). This same study indicated substantial astrocyte loss in mixed midbrain cultures derived from the same mouse line (36). In contrast, other studies did not identify a change in astrocyte reactivity (39), microglial activation (41) or overall numbers of these glial cells (15, 33). Therefore, there is limited evidence linking exogenous GDF15 modulation to glial neuroinflammatory responses following induced neurodegeneration.

### 3.6 Elevated GDF15 is associated with neurodegenerative disease and injury

The second aim of this systematic review was to assess GDF15 levels as a biomarker in individuals with neurodegenerative disease or injury. Overall, the studies examined show that GDF15 levels are elevated in some neurodegenerative conditions and are associated with disease severity and incidence (**Table 9**; **Supplementary Table 2**).

**Table 9:** Measuring GDF15 expression levels in models of neurodegeneration.

Pathophysiology	Neurodegenerative Agent	Neurodegenerative Condition/Phenotype	Type of model	Tissue/fluid measured	Change in GDF15 relative to control	Ref
**DNA damage**	Etopside	DNA damage	*In vitro* (primary astrocytes)	Cell lysate, media	Increase	(49)
**Excitotoxicity**	Kainic acid	Excitotoxicity	*In vivo* (mouse, ICR)	Tissue (hippocampus CA3), cells (astrocytes)	Increase	(47)
**Genetic**	Transgenic (APP/PS1)	AD	*In vivo* (mouse, APP/PS1)	Brain (unspecified)	Decrease	(30)
	Transgenic (GARS)	CMT (2d)	*In vivo* (mouse, GarsP278KY/GarsC201R)	Serum	Increase	(50)
	Transgenic (Gjb1)	CMT (1x)	*In vivo* (mouse, Gjb1 null)	Serum	Increase	
	Transgenic (Hspb8)	CMT (2L)	*In vivo* (mouse, HSPb8(K141N) KI)	Serum	Increase	
	Transgenic (PMP22)	CMT (1a)	*In vivo* (mouse, C61 het)	Serum	No change	
	Transgenic (Gpnmb, Tyrp1)	RD	*In vivo* (mouse, DBA/2J)	Retina, aqueous humour	Increase	(51)
	Transfection, HTT-mutant	HD	*In vitro* (PC-12)	Cell lysate	Increase	(42)
	Transfection, PMPP overexpression	CMT	*In vitro* (RT4, WJ-MSC coculture)	Cell media	Increase	(27)
	Transgenic (SMN)	SMA	*In vivo* (mouse, SMN^-/-^ SMN2^+/+^ SMDδ7^+/+^)	Cells (oculomotor, trochlear somatic motoneurons)	Increase	(40)
	Transgenic (R19H|HO and 132H|HO)	VWMD	*In vivo* (mouse, R19H|HO and 132H|HO)	Cerebellum, spinal cord, CSF	Increase	(52)
	Transgenic (R19H|HO and 132H|HO)	VWMD	*In vivo* (mouse, R19H|HO and 132H|HO)	Plasma	No change	
**Inflammation**	Experimentally induced uveitis (LPS)	RD	*In vivo* (mouse, C57BL/6J)	Retina	No change	(51)
	IL-6	Neuroinflammation	*In vitro* (PC-12)	Cell lysate	Increase	(53)
	LPS	Neuroinflammation	*In vitro* (primary astrocytes)	Cell lysate	Increase	(47)
	Optic nerve homogenate antigen	RD	*In vivo* (rat, Lewis)	Retina	Decrease	(54)
	TG-rich lipoprotein, lipoprotein lipase	Neuroinflammation	*In vitro* (astrocytes)	Cell lysate, media	Increase	(48)
**Integrated Stress Response**	Tunicamycin	VMWD	*In vitro* (primary astrocytes)	Cell media	Increase	(52)
**Mechanical**	Cryolesion	CNS Injury	*In vivo* (rat, Wistar)	Tissue (lesion)	Increase	(55)
	Middle cerebral artery occlusion	Stroke	*In vivo* (mouse, C57BL/6)	Tissue (hippocampus), cells (neurons, microglia - not astrocytes)	Increase	(56)
	Optic nerve crush	RD	*In vivo* (mouse, C57BL/6J)	Retina, aqueous humour	Increase	(51)
	Optic nerve crush	RD	*In vivo* (rat, Sprague Dawley)	Retina, aqueous humour	Increase	
	Light induced retinal degeneration	RD	*In vivo* (mouse, 129S1/SvlmJ)	Retina	No change	
	Optic nerve crush	RD	*In vivo* (mouse, C57BL/6J)	Retina	Increase	(26)
	Optic nerve crush	RD	*In vivo* (mouse, ddY)	Retina, optic nerve	Increase	(37)
	Optic nerve crush	RD/VWMD	*In vivo* (mouse, C57BL/6j)	Retina	Increase	(52)
	Sciatic nerve crush/transection	PNS Injury	*In vivo* (mouse, GDF15 and Wt)	Tissue (DRG, distal nerve)	Increase	(43)
**Mitochondrial dysfunction**	6-OHDA	PD	*In vivo* (mouse, Wt)	Tissue (CPu, SN)	Partial increase	(36)
	MPTP	PD	*In vivo* (mouse, Wt)	Tissue (CPu, SN)	Increase	(39)
	MPP	Mitochondrial dysfunction	*In vitro* (mesencephalic cells)	Cells	Increase	(57)
	Rotenone	MS	*In vitro* (astrocytes, HCPEpiC choroid plexus epithelia)	Cell media	Increase	(58)
	Rotenone	MS	*In vitro* (primary neurons, HCMEC/D3 brain endothelia,CHME5 microglia)	Cell media	No change	
	Rotenone	PD	*In vitro* (SH-SY5Y)	Cell lysate, media	Increase	(28)
	Transfection, pEGFP-FTMT overexpression	Mitochondrial dysfunction	*In vitro* (SH-SY5Y)	Cell lysate	Increase	(59)
**Pathogenic peptide**	Aβ25-35	AD	*In vitro* (SH-SY5Y)	Cell lysate	Decrease	(30)
	Aβ42	AD	*In vitro* (BV2, h-UCB-MSC coculture)	Cell lysate	No change	(34)
	Aβ42	AD	*In vitro* (BV2, h-UCB-MSC coculture)	Cell media	Increase	
**Phenotypic (patient-derived)**	n.a	AD	*In vitro* (fibroblasts)	Cell lysate, media	Increase	(35)
	n.a	CNS Injury	*In vitro* (mesenchymal stromal cells)	Cell lysate	Increase	(60)
	n.a	ALS (CHCHD10 p.15L)	*In vitro* (fibroblast)	Cell lysate	Increase	(61)

#### 3.6.1 Mitochondrial disease

Mitochondrial diseases are caused by mutations in mitochondrial DNA (mtDNA) and mitochondrial genes (62) and show consistent upregulation of GDF15. As a secreted protein, GDF15 circulates in blood in a normal range of (200 – 1200 pg/mL) (11). Across paediatric and adult cases, blood levels of GDF15 were elevated in mitochondrial disease patients compared to their healthy counterparts (63–73). Two of these studies found that this elevation related to neurodegenerative outcomes. The first highlighted an association between GDF15 levels and supratentorial grey matter atrophy and white matter microstructural changes identified by magnetic resonance imaging (MRI) (74). The second showed that this relationship is further strengthened by stratifying cases into mitochondrial encephalomyopathy with lactic acidosis and stroke-like episodes and myoclonic epilepsy with ragged red fibres (65). However, this was not replicated in a broader cohort (70), and remains unclear. Although GDF15 is associated with aging, neurodegeneration and all-cause mortality more generally, it should be noted that increased levels were not associated with age in mitochondrial disease (63, 65, 67, 68, 75), with one weak exception in paediatric cases (70). This suggests that GDF15 elevation in these conditions is not an age-associated phenomenon, rather, reflective of an upregulated mitochondrial stress response. Indeed, mitochondrial disease patients had higher circulatory GDF15 levels compared to non-mitochondrial neuromuscular disease controls (63, 64, 68–71). Further, several studies utilized mitochondrial dysfunction to induce a neurodegenerative phenotype *in vitro* and *in vivo*, which caused elevated GDF15 expression and secretion (28, 36, 39, 57–59). Therefore, heightened circulatory GDF15 indicates mitochondrial stress and dysfunction, likely associated with neurodegenerative pathophysiology.

#### 3.6.2 Alzheimer’s disease and related dementias

Upregulated blood GDF15 was further revealed to be associated with risk of dementias, neurodegenerative diseases affecting cognition, including AD. AD is characterized by progressive neuron and synapse loss, alongside accumulation of amyloid-β (Aβ) plaques and hyperphosphorylated Tau neurofibrillary tangles (35). With respect to relative levels in plasma, GDF15 was increased in AD (76) and dementia patients (77) compared to individuals with no cognitive impairment. A significant increase was also reported in individuals with mild cognitive impairment (76–78). In contrast with blood GDF15, CSF levels did not vary in AD patients compared to controls (35). In assessing risk, one study performed 2-sample Mendelian randomization analysis using summary-level datasets from GWAS for an AD population. The authors identified that increased serum GDF15 levels was associated with higher AD risk (79). This relationship was further evident in assessing prospective cohorts for AD and dementia incidence (80–84), although significance was lost for AD when including N-terminal pro-brain natriuretic peptide (NT-proBNP), a marker of ventricular distention (80). Another study found a correlation between elevated GDF15 and AD Pattern Similarity (AD-PS) score, an index of neuroanatomical dementia risk that compares grey matter spatial patterns (85). Consistent with this, other examinations showed increased peripheral GDF15 was associated with white matter hyperintensities (76) and that this was strengthened in cohorts with cognitive impairment relative to cognitively normal individuals (78).

In addition to its potential as an AD blood biomarker, dysregulation of central GDF15 was reported, albeit with inconsistent findings. Firstly, increased GDF15 was identified in multiple post-mortem AD brain areas (86). This finding conflicted with another study that reported no change in GDF15 levels in post-mortem frontal cortex tissue (35). Although, this study did find an increased ratio of mature to precursor GDF15 in AD patients and centenarians. Given that processed, mature GDF15 rapidly diffuses away into circulation (6), this change may reflect an age or disease-associated increase in GDF15 processing. However, dermal fibroblasts taken from AD subjects expressed significantly more GDF15 mRNA and secreted more protein into media than age-matched non-demented subjects (35). This incongruity implies that increased GDF15 is more closely associated with Alzheimer’s pathology than with healthy aging. Additionally, BV2 microglia secreted more GDF15 into media with MSCs when incubated with Aβ42 (34), suggesting GDF15 upregulation is triggered by AD pathology to attenuate pathogenic mechanisms. On the other hand, one study found that GDF15 mRNA and protein was reduced in both the brain of a familial AD mouse model compared to wildtype controls, and in SH-SY5Y cells incubated with Aβ 25-35 peptide (30). These inconsistencies in GDF15 expression may relate to differences in microglia and neuronal responses to Aβ, as well as variance between familial and late onset AD pathology, although these effects are not compared experimentally. Ultimately, whilst GDF15 upregulation in AD pathogenic models and *ex vivo* tissue is inconsistently demonstrated, it may have potential utility as a biomarker for AD and other dementias.

#### 3.6.3 Synucleinopathy

Synucleinopathies represent a class of neurodegenerative diseases pathologically characterized by aggregated and phosphorylated α-synuclein. These diseases include Parkinson’s disease (PD), PD with dementia (PDD), Dementia with Lewy Bodies (DLB) and Multiple System Atrophy (MSA) and vary clinically by variation in region and cellular pathology. Two studies identified that serum GDF15 was elevated in PD cases compared to controls (66, 87), whilst another showed no statistical difference (88). One study examined MSA patients (89), demonstrating elevated serum GDF15 relative to both healthy controls and PD. Further, GDF15 levels were higher in cases with longer disease duration, older age, and MSA-Parkinsonian subtype. A sex effect was also uncovered, with higher serum GDF15 in male synucleinopathy patients relative to females (87, 89). With respect to CSF measurements, GDF15 was increased in all Lewy body diseases, particularly for the PDD subgroup (90). Older age at symptom onset was related to higher GDF15 in two studies (88, 90) but not in another (87). Furthermore, elevated serum GDF15 was associated with disease duration and worsened motor and cognitive scores and was reflective of poorer neurological outcomes in these synucleinopathy cohorts (87, 90). Further, measures of neuronal death and axonal damage t- and p-Tau correlate with GDF15 levels in CSF (90). A recent longitudinal assessment of a large preclinical group showed plasma GDF15 levels as associated with higher risk for PD (82), whilst an earlier study did not identify this risk (79). Induction of parkinsonian-like pathology in cell and animal models was also found to trigger upregulated GDF15. Use of inflammatory neurotoxins increased GDF15 expression in the caudate putamen and substantia nigra of mice (36, 39), and in both cell lysate and media of SH-SY5Y neurons (28). Thus, there is some support for increased serum GDF15 in synucleinopathy, and this elevation may be an intrinsic component of synuclein pathogenesis.

#### 3.6.4 Multiple sclerosis and neuromyelitis optica spectrum disorder

MS is a chronic neuroinflammatory disorder causing demyelination and neuron degeneration in the entire CNS. MS is further categorized into clinical phenotypes that include relapsing-remitting (RRMS), characterized by acute and highly variable attacks; primary progressive (PPMS), which gradually worsens from onset; and secondary progressive (SPMS), which initially presents as RRMS and progresses to resemble PPMS later in disease development (91). Studies that do not differentiate between MS cases fail to detect any differences in serum GDF15 relative to healthy controls (72, 92). Interestingly, this is not the case when studies examine the specific clinical phenotypes of MS. Individuals diagnosed with PPMS or SPMS had elevated GDF15 in serum and CSF relative to control (58, 93) and RRMS cases (58). No significant increase was observed between RRMS and controls in these studies (58, 93).

Given the heterogeneity in MS cases, it is therefore unsurprising that altered levels of GDF15 was found to relate to clinical course and severity. In cohorts with undefined MS, serum GDF15 was elevated 7.4 months into disease progression (92), and positively associated with Expanded Disability Status Scale (75). However, this could not be replicated in a purely PPMS group (93). In fact, baseline GDF15 was reduced in PPMS patients who had worsened motor outcomes at 18 months (93). Contrastingly, GDF15 levels were higher in RRMS patients who did not develop gadolinium-enhancing or T2 lesion activity, indicative of focal inflammation (91, 94). When put together, these results suggest that the overall pattern of GDF15 dysregulation in MS is possibly linked to both clinical and pathological phenotypes and may be reflective of underlying neuroinflammation. Future studies examining GDF15 as an MS biomarker should substantiate these findings by directly relating the disease subtype.

Further to fluid biomarker examination of GDF15 levels in MS, there is some support for the role of astrocytes in the production of this cytokine to mediate neuroinflammatory responses. One study found 1 – 10% of reactive astrocytes and macrophages/microglia expressed GDF15 immunoreactivity in demyelinating lesions localized to the frontal cortex of a patient with SPMS (49). Cell models recapitulating MS pathology have shown that astrocytes express and secrete greater GDF15 in response to oxidative stress (49) and mitochondrial dysfunction (58). This response is likely to both induce neurotrophic effects in damaged neurons and signal cell stress associated with MS lesions.

Research examining serum GDF15 in individuals with Neuromyelitis Optica Spectrum disorder, a demyelinating autoimmune condition akin to MS, was also included in this review. No significant differences were shown between patient and control cases (67, 72), suggesting it is not a suitable biomarker for this disease and potentially reflecting variation from MS pathophysiology.

#### 3.6.5 Glaucoma

Glaucoma refers to a group of neuroretina degenerative diseases associated with retinal ganglion cell death. Studies included in this review assessed GDF15 in the context of biomarkers for primary open angle glaucoma and pseudoexfoliative glaucoma, showing elevated levels in both diseases (51, 95). Further, this elevation in GDF15 appears to be unrelated to surgical interventions, with one study showing some patients have decreased and others increased GDF15 post-operatively (96). In rodent models, GDF15 expression changes were largely dependent on the type of model used. GDF15 was upregulated both in the retina and aqueous humor of chronic glaucoma mouse models at 1 year (51). Further, optic nerve crush also elevated GDF15 levels in mice (26, 37, 52) and rats (51) in 1 – 9 dpi. In contrast, no change was observed at any timepoint in the mouse retina following light-induced retinal degeneration or in experimentally induced uveitis (51). Moreover, 28 days after systemic injection of optic nerve homogenate antigen, mRNA expression of this cytokine was diminished in the inner retinal layer (54). Therefore, while GDF15’s association with retinal degeneration varies, fully understanding its role in neuroretina inflammation requires further evidence and additional timepoints.

#### 3.6.6 Motor neuron disease

Research examining GDF15 dysregulation in MNDs is limited, with conflicting support between human and animal/cell-based studies. With respect to ALS, serum GDF15 was not associated with disease risk (79). However, fibroblasts derived from an ALS patient with a CHCHD10 p.15L gene variant were shown to express more GDF15 than the wildtype line (61). Similarly, one study showed no significant serum difference between Spinal Muscular Atrophy Jokela type individuals and healthy controls (97). On the other hand, in a mouse model of this condition, examination of motoneurons that are resilient to degeneration showed differential upregulation of GDF15 alongside other anti-apoptotic factors (40). Thus, although there is insufficient evidence to assert if it acts as a suitable biomarker for either MND, GDF15 may be associated with their underlying neuropathogenic processes either by reflecting cell stress or promoting cell survival pathways.

#### 3.6.7 Charcot-Marie-Tooth disease

CMT is a type of demyelinating, peripheral nervous system neuropathy. One study determined serum GDF15 was elevated across all subtypes of this condition and was worsened by disease severity as measured by the progression score CMTES (50). A recent study indicated GDF15 did not vary between genetic variants CMT1a and PMP22 for the disease (98). This is supportive of findings from mouse models of CMT that show GDF15 was elevated in serum (50) and in schwannoma cell media when transfected with PMP22 (27).

#### 3.6.8 Huntington’s disease

HD is a complex neurodegenerative disorder with psychological, cognitive, and motor symptoms. Pathologically, this disease is driven by mutant HTT fibrillation and multimerization. Our review did not identify any HD biomarker studies, although the HTT mutant was shown to lead to higher levels of GDF15 than control transfected neurons (42).

#### 3.6.9 Vanishing white matter disease

VWMD is a progressive hypomyelinating disease caused by bi-allelic variants in eukaryotic initiation factor 2B, which mounts the integrated stress response. One included publication examined VWMD in human populations, with complementary *in vivo* and *in vitro* modelling (52). Elevated GDF15 was reported in VWMD patient CSF compared to controls, but not in plasma or serum. This was consistent with VWMD mouse models, which additionally exhibit increased GDF15 expression in the cerebellum and spinal cord at 4 months relative to wildtype. Upregulated GDF15 was determined to be astrocytic in origin; GDF15 transcripts were localized to affected mouse forebrain astrocytes, and increased secretion occurred in response to tunicamycin *in vitro* (52). In summary, this study reveals a significant association between VMDW and elevated GDF15, mediated by the integrated stress response.

#### 3.6.10 Neurodegeneration secondary to injuries to the central and peripheral nervous system

Studies examining GDF15 in the context of secondary neurodegeneration due to neurotrauma were also assessed in this review. One key finding was that circulatory GDF15 is closely related to the incidence, severity, and risk of stroke. Increased GDF15 in blood was observed in the acute phase following an ischemic event in affected individuals relative to healthy controls (99–101). It was also evident that blood GDF15 at time of admission was consistently elevated in stroke patients who later displayed poorer modified Rankin Scores (mRS) both at discharge and 90 days follow up (102, 103). Elevated blood GDF15 was furthermore associated with injury/inflammation biomarkers glial protein S100 calcium binding protein B and IL-6 (99, 102) and with lesion size and severity (99, 100, 103). Interestingly, GDF15 on admission correlated with depression scores at 90 days and was predictive of post-stroke associated depression (99). Longitudinal studies linked blood GDF15 levels with the risk of incident stroke/TIA, although there was some evidence this was related to cardiovascular events (82, 101, 104, 105).

There is some evidential support for central as well as peripheral expression of GDF15 in response to ischemic stroke. Increased GDF15 staining was identified in a small subset of reactive astrocytes and macrophages/microglia in post-mortem ischemic lesions following acute cerebral infarction (49). Furthermore, GDF15 expression and immunoreactivity was also elevated in mouse hippocampus 3 – 24 h following middle cerebral artery occlusion (56). Together with blood biomarker findings highlighted above, these studies indicate that GDF15 serves as a putative stroke biomarker, although future work should elaborate further on the source of centrally induced GDF15.

Besides stroke, examination of GDF15 in neurotrauma-induced degenerative conditions in human populations was limited. One study showed that serum GDF15 was elevated in patients following subarachnoid haemorrhage, and this elevation in the first 9 days related to worsened primary and secondary neurological outcomes at 90 day follow up (106). Another study examined mesenchymal stromal cells derived from individuals with SCI or traumatic brain injury (TBI). The authors identified increased GDF15 secretion into cell lysate, indicative of a widespread cell stress response (60). This induction was also reported in injured rodent CNS and PNS tissue in response to cryolesion (55) and sciatic nerve crush (43) respectively. Overall, although limited in number and scope, these studies highlight that increased expression of GDF15 can be identified in mixed neurotrauma states.

## 4 Discussion

To better understand GDF15’s role in neurodegeneration, we conducted a systematic review of the literature. Our review explored the possible mechanisms underlying GDF15’s neuroprotective function in animal and cell models, as well as its potential as a biomarker for neurodegenerative conditions. Overall, the included studies showed that exogenously increasing GDF15 is generally neuroprotective, whilst silencing/knockdown is linked to largely exacerbated neurodegeneration. Favorable outcomes related to GDF15 modulation were attributable to its anti-apoptotic effects, increased cell viability, alleviation of mitochondrial and oxidative stress, and modulation of neuroinflammation. Furthermore, included biomarker studies support a recent meta-analysis highlighting the elevation of GDF15 in select studies examining AD, PD and MSA (107). This present review expands that work by showing that GDF15 levels were higher in individuals with most chronic neurodegenerative conditions, and, with the exception of MS, these elevated levels were typically associated with increased disease incidence and severity. Together, this highlights the potential utility of GDF15 as both a therapeutic tool and a possible biomarker in neurodegeneration.

One key finding from this review is that despite the multitude of GDF15-induced effects observed, there is a lack of studies showcasing these via interactions with its target receptor GFRAL. In fact, this review identified only one citation demonstrating neurotrophic activity mediated by GDF15-GFRAL-RET in neurons (37). However, several *in vitro* studies detail how altering GDF15 impacts apoptotic pathways, cell viability, bioenergetic stress and cytokine expression in neuron only cultures (24, 28, 30, 31). This suggests that GDF15 can elicit protective effects on neurons directly, regardless of an apparent lack of specific receptor. As GDF15 can also modulate cell types which affect neuronal survival, some studies point towards GDF15 exerting indirect effects on neurons. For instance, GDF15 silencing in astrocytes worsened dopaminergic neuron loss (38) likely due to induced astrocyte cell loss (36), therefore reducing neurotrophic support. Included studies further showed that perturbed peripheral immune cell recruitment (15, 43) and microglia proliferation (36) is linked to GDF15 modulation. The potential mechanisms of action underlying GDF15’s directly or indirectly mediated effect is presently discussed.

### 4.1 GDF15’s neuroprotective role is likely mediated centrally by GFRAL-RET

Despite the emphasis on the activation of the PI3K/Akt/ERK pathway as a potential downstream pathway of GDF15, it remains unclear how GDF15 triggers this activation in neurodegeneration. Prior to identifying GFRAL as its target receptor, the literature primarily attributed these responses to TGF-β family receptors I (TGF-βI) and II mediated Smad pathway phosphorylation. However, there is no strong evidence to show that these receptors are activated by GDF15 (10), as studies show this could be inadvertently reporting TGF-β activity due to contamination of commercial GDF15 preparations (12). Our review highlights a lack of studies investigating the mechanisms of GFRAL-mediated neuroprotection, possibly due to the limited areas of expression identified for this receptor. It is possible that GFRAL expression is more widespread than currently established, or is inducible by disease, but is not detectable by standard laboratory methods (11). Alternatively, as the main alternate transcript of GFRAL lacks a transmembrane sequence (108), it could serve as a soluble receptor (11). It is possible that GDF15 binds this soluble form, enabling RET engagement in cells that do not typically express GFRAL (109). This mechanism bears similarity to that of IL-6 trans-signalling, in which soluble IL-6R can bind to co-receptor gp130, making virtually all cells responsive to IL-6 (110). It is well established that RET tyrosine phosphorylation activates MAPK/ERK, PI3K/Akt and Rac/cJun JNK pathways that are anti-apoptotic (108) and drive cellular proliferation, differentiation, functioning and more (reviewed in 111, 112). One study in our review showed that a RET inhibitor suppressed Akt activation and therefore diminished neurotrophic outcomes attributed to rGDF15 supplementation *in vitro* (37). Trans-signalling could account for the discrepancy between the widespread induction of GDF15 and limited GFRAL expression, however, other receptors or modes of activation cannot be ruled out. For instance, GFRAL knockout mice have similar body weight, fat, and mass to wildtype mice, highlighting the possible presence of non-GFRAL mediated GDF15 activity (113), though germline GFRAL knockout may cause other compensatory effects. Alternatively, GDF15 may competitively inhibit activation of receptors that mediate harmful effects. For example, GDF15 hinders recruitment of polymorphonuclear leukocytes into myocardial infarcts and may limit occurrence of cardiac rupture (16). Lastly, we cannot rule out the possibility of indirect induction of TGF-β signalling through GDF15-microRNA interaction (114), or through uncharacterized, non-GFRAL mediated activity in immune cells (115, 116). However, future studies on GDF15 should prioritize mechanistic investigations of GFRAL-RET signalling in neurodegeneration.

### 4.2 GDF15 deficiency may activate compensatory immunomodulatory effects that drive cellular regeneration in neurodegeneration

Our review revealed a disparity in the immunomodulatory effects observed when GDF15 levels were experimentally raised or lowered in neurodegenerative models, with implications for cellular regeneration and functional recovery. GDF15 overexpressing SCI mice had increased infiltration of macrophages and dendritic cells to SC, as well as improved pathology resolution and motor outcomes (15). This suggests that macrophages may aid in myelin debris clearance (15), while dendritic cells recruit regulatory T-cells that alleviate pathology (117). This favorable effect is reflected in human MS biomarker cohorts, where elevated peripheral GDF15 is associated with improved neuropathological (94) and motor outcomes (93). Unlike in other neurodegenerative conditions where elevated GDF15 correlates with greater disease severity, this may indicate that certain MS individuals reflect a transient stress response, and GDF15 may effectively resolve these effects in these cases. In contrast with increased GDF15, silencing or knocking down GDF15 did not consistently potentiate tissue damage or functional deficits associated with induced injury or disease. GDF15 null mice did not have worsened tissue damage (15), nerve damage or functional output (15, 43). This asymmetry could stem from the compensatory increase in IL-6 levels observed when GDF15 is depleted (29, 30, 36, 43). IL-6 regulates anti-inflammatory as well as pro-inflammatory responses, as evidenced by its ability to alleviate pathogenic changes in vascular injury (118) and in myelin antigen triggered neuroinflammation (119). Elevated IL-6 levels in GDF15 null mice following peripheral nerve injury may account for why worsened axon loss and demyelination was not reported here (43). Contrastingly, increased GDF15 diminishes IL-6 and alleviates Aβ induced impairments by inhibiting neuronal apoptosis *in vitro* (30). Therefore, IL-6 signalling may compensate for GDF15 deficiency, while elevated GDF15 may minimize pro-inflammatory IL-6 responses; however, due to limited and inconsistent studies on this pattern, our conclusions remain speculative and other beneficial immune mediated responses may be involved.

### 4.3 The U-curve dose-response relationship of GDF15 in neurodegeneration

**Figure 2:**
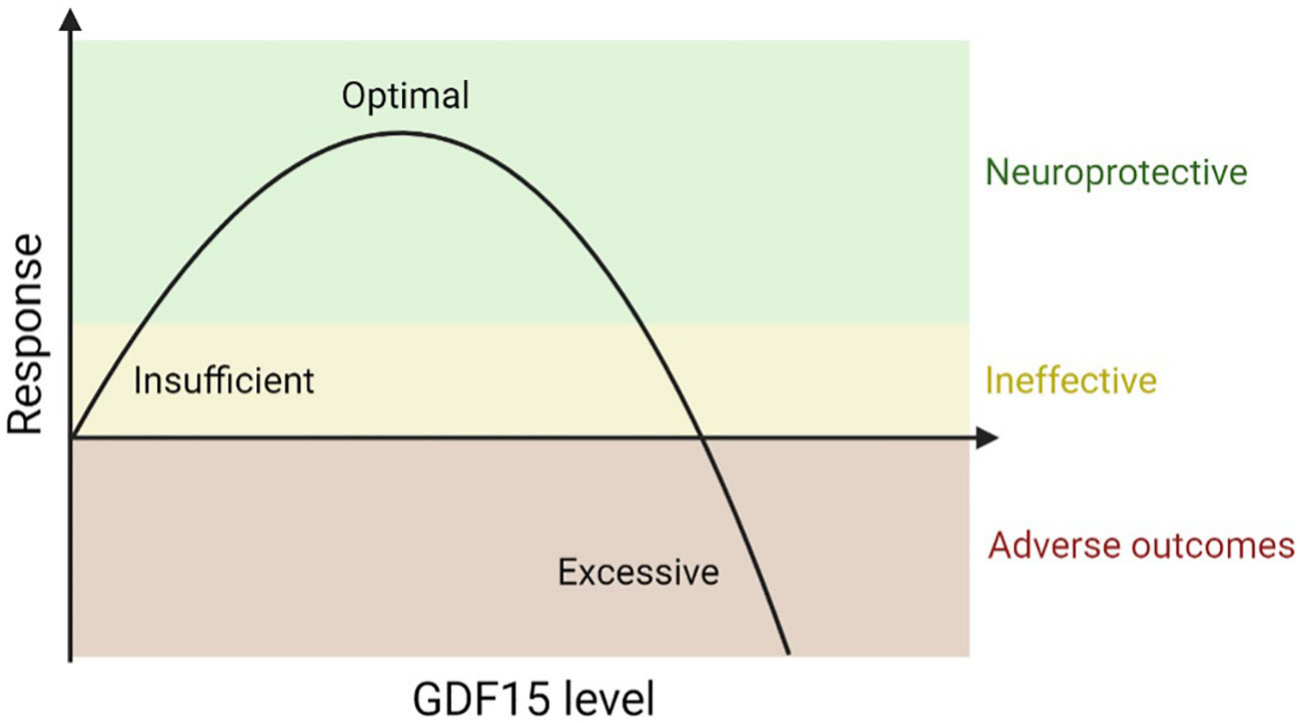
Proposed Inverted U-Curve Dose-Response Relationship of GDF15 to Neurodegeneration. This schematic illustrates the dose-response relationship between GDF15 levels and neurodegenerative outcomes. Low level, basal GDF15 has minimal impact on neurodegeneration. An optimal, intermediate level of GDF15 promotes therapeutic effects and neuroprotection. High levels of GDf15 indicate excessive cell stress and is associated with adverse neurodegenerative effects.

Our review highlights that GDF15 acts as a neuroprotective agent in disease and injury models, yet its elevation in some patients is linked to poorer outcomes. This is consistent with existing literature on GDF15’s role in exercise and cancer, demonstrating a U-shaped dose-response to stress. Vigorous endurance exercise, which induces transient mitochondrial stress, spikes plasma GDF15 comparable to patients with MD, infection, and cancer (113, 120). This increase in GDF15 is also observed in mice; however, unlike the effect of high-dose exogenous delivery of rhGDF15, it does not suppress appetite and running activity (113). Higher endogenous GDF15 is also observed in chronically inactive and aging populations (120) and is inversely associated with survival (121). Furthermore, while GDF15 offers protection by alleviating impaired mitochondrial function, its induction of the mitochondrial integrated stress response in papillary thyroid carcinoma patients can also contribute to cancer progression via the GDF15-STAT3 pathway (122). Therefore, optimal levels of GDF15 exhibit protective and therapeutic effects, but elevated or sustained levels in specific pathogenic contexts indicate insurmountable cellular stress and serve as disease biomarkers (**Figure 2**). Crucially, elevated GDF15 observed in disease models and in epidemiological studies does not imply disease causality. As proposed by Breit and colleagues, it is possible that while neurodegeneration induces GDF15 in proportion to disease severity as a reparative response, the prolonged elevation of GDF15 eventually becomes inadequate in alleviating or reversing disease-related stress (11, 109). This inverted u-curve relationship may explain why chronically induced endogenous GDF15 in affected individuals fails to lessen aspects of neurodegeneration that are more effectively addressed in cell and animal models. Supporting this, one included study showed that administering 2Bact, an inhibitor of the integrated stress response, to Vanishing White Matter disease and optic nerve crush mice resulted in reduced GDF15 levels and improved pathology (52). Future investigations should prioritize a qualitative analysis of optimal GDF15 levels in neurodegenerative settings.

### 4.4 Limitations of this review and recommendations for future work

It is important to highlight that the broad scope of this review may limit the conclusions drawn. Firstly, we did not incorporate specific neurodegenerative disorders like Alzheimer’s or Parkinson’s disease into our database search. Consequently, some relevant studies such as (123) may have been overlooked, although this is likely only a sporadic occurrence given our search strategy (including search terms GDF15 and its synonyms) was inclusive of titles and abstracts. Nonetheless, our aim was to evaluate neurodegeneration in a general context, rather than specifically investigating the role of GDF15 within distinct disease or trauma states. Secondly, the literature spanned in this review included highly variable animal and cell studies. This variability extends to factors like method of induced neurodegenerative disease or injury, method of GDF15 modulation, types of outcomes measured, spatial and temporal assessment and animal age, all impacting functional outcomes. For instance, one study shows significant motoneuron loss in GDF15 null mice at 6 months but not at P14 or 3 months (41). This suggests many facets of disease or injury-related responses may not be accounted for in our assessment. Despite this, the consistency in findings across these studies underscores the robustness of GDF15 as a neuroprotective factor. Lastly, risks associated with pharmacological administration of GDF15 were not extensively considered and may impact translation of GDF15 therapeutics into clinical settings. In depth investigations should be undertaken to identify how to best balance the benefits of GDF15 supplementation against its risks, including anorexia/cachexia and weight loss (11, 109). Further, doses administered in preclinical studies raise GDF15 levels well beyond normal physiological conditions (113), questioning their relevance. Future research should consider human-equivalent dosing for more translatable findings.

Results drawn from relevant biomarker studies also have important considerations. Firstly, the included studies may underreport GDF15 levels in individuals with homozygous and heterozygous H6D variants (124). Unfortunately, our review lacks a straightforward solution to this issue, as it necessitates a direct performance comparison of all available immunoassays. As a result, there is a possibility that many of these studies report inaccurate intergroup differences. This highlights a critical need for biomarker studies to either utilize assays that specifically accommodate for this H6D variant or incorporate suitable statistical models to address variability. Furthermore, the use of prospective cohorts in some studies posed challenges in distinguishing GDF15-related disease risk directly associated with neurodegeneration as opposed to risks associated with all-cause mortality (22), infection (125) or cardiovascular disease (126). Notably, certain studies lost significance when adjusting for cardiovascular risk factors (80, 104), indicating that cardiovascular outcomes may play a significant role in driving this relationship. Therefore, considering the widespread induction of GDF15 across various biological systems and in aging, it is improbable for it to serve as a suitable biomarker with single level performance. GDF15’s utility is improved when used in combination with clinical data or additional blood biomarkers (84). Although, repeated measures within an individual may still be useful to assess treatment activity wherever available (127–129).

## 5 Conclusion

Neurodegeneration represents a complex phenomenon characterized by limited treatment options to impede or decelerate its progression. One of the primary pathophysiological mechanisms implicated in these disorders is neuroinflammation, prompting growing clinical interest in immunomodulatory agents. GDF15 is an anti-inflammatory cytokine with established neurotrophic properties. The evidence outlined in this review suggests that GDF15 expression is induced in response to neurodegenerative pathophysiology and inducing higher levels yields favorable outcomes. Nevertheless, further preclinical investigations which address existing methodological pitfalls are imperative to support these findings mechanistically. Addressing these gaps will facilitate an enhanced understanding of the neuroprotective mechanisms mediated by GDF15, thereby paving the way for novel therapeutic interventions.

## Data Availability

The original contributions presented in the study are included in the article/**Supplementary Material**. Further inquiries can be directed to the corresponding author.
